# *Aspergillus fumigatus* Epidural Abscess and Postsurgical Wound Infection in an Immunocompetent Host

**DOI:** 10.1155/2024/8104167

**Published:** 2024-02-29

**Authors:** Nadine Montreuil, Andres Martinez, Leon Budrie, Shriya Goyal, Tanya Quiroz, Christine Vu, Folusakin Ayoade, Candice A. Sternberg

**Affiliations:** ^1^University of Miami, Miller School of Medicine, Miami, FL, USA; ^2^Jackson Health System, Miami, FL, USA; ^3^Baylor College of Medicine, Houston, TX, USA; ^4^Louisiana State University, Shreveport, LA, USA

## Abstract

In this case, we present an immunocompetent patient who had a wound infection secondary to *Aspergillus fumigatus* after undergoing a neurosurgical procedure that was complicated by an epidural abscess. The patient was treated with voriconazole and responded favorably. We highlight the need for awareness of the possibility of an *Aspergillus* infection in people without any obvious immunocompromise and advocate for the inclusion of this opportunistic fungus in the workup of postneurosurgical infections and dura-based collections. A brief review of relevant literature is also included.

## 1. Introduction

Aspergillosis is an opportunistic fungal infection that is caused by *Aspergillus*, which is ubiquitous in the environment. Most cases of central nervous system (CNS) *Aspergillus* infections are seen in patients who are immunocompromised such as transplant patients, patients with cancer, on chemotherapy, or prolonged steroids [1]. In addition to immunocompromised status, other factors that can also increase the risk of infection include prior surgeries and any steroid use, even when temporary and of short duration [2–4]. Although invasive *Aspergillus* infections are usually seen in patients who are immunocompromised, immunocompetent individuals can also be affected by aspergillosis postsurgery, which remains rare. In fact, most cases of reported postsurgical aspergillosis are encountered in immunocompetent individuals [4–6]. Here, we present a case of an immunocompetent patient who developed an *Aspergillus* epidural abscess after an elective neurosurgical procedure despite no previous steroid exposure. The clinical presentation of aspergillosis epidural abscess is nonspecific and may mimic other conditions such as an abscess due to bacterial or mycobacterial pathogens, and therefore, diagnosis can be challenging and often delayed [7]. This case report aims to raise awareness about postsurgical complications with *Aspergillus* CNS infections in immunocompetent hosts and highlights the importance of timely diagnosis and management. We also include a brief review of relevant literature findings.

## 2. Case Presentation

A 54-year-old African American female with a past medical history of hypertension, deep vein thrombosis (not on anticoagulation), and recently diagnosed Chiari malformation with syringomyelia presented with new-onset headaches and fevers.

She reported a pressure-like throbbing headache associated with body aches, subjective fevers of 2 days duration, as well as numbness in her left foot, neck pain with movement, and increased redness and pain around the surgical site.

Three weeks prior to presentation, the patient underwent elective neurosurgery to correct a Chiari malformation with syringomyelia. A suboccipital craniectomy with C1 laminectomy and duraplasty with cadaveric dura was performed at this admission without complications, and the patient was discharged home.

At the time of presentation, the patient was hemodynamically stable, saturating 97% on room air, with low-grade temperature. On physical exam, she appeared ill. There was tenderness in the neck area, especially with neck flexion. The posterior neck incision appeared clean, dry, and well-healing. The rest of the physical exam was without pertinent findings.

**Figure 1:**
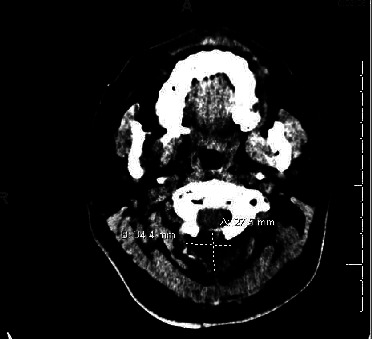
CT imaging (transverse section) showing surgical site collection including a pseudomeningocele and epidural abscess.

**Figure 2:**
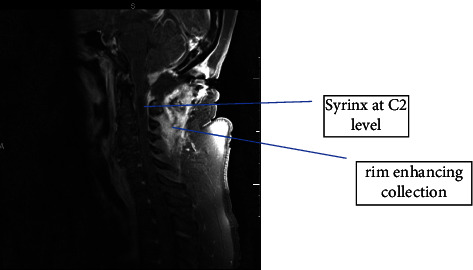
Sagittal section of MRI brain with/without contrast showing a rim-enhancing collection and 2.3 cm × 0.9 cm syrinx at the level of C2.

Initial workup included a normal peripheral blood leukocyte count, negative rapid HIV screen, and a basic metabolic panel without electrolyte derangements. The computed tomography (CT) of the brain showed no signs of a stroke or bleeding, but findings were consistent with a recent craniectomy with C1 laminectomy and duraplasty. CT also showed a fluid collection measuring 3.4 cm × 2.8 cm (Figure 1). Follow-up magnetic resonance imaging (MRI) showed a rim-enhancing collection likely contiguous with cerebrospinal fluid (CSF) spaces and 2.3 cm × 0.9 cm syrinx at the level of C2 (Figure 2).

Based on these findings, the neurosurgical service took the patient emergently to the operating room for an incision and drainage of the collection. At the time of surgery, a seroma, as well as an abscess, was found deep in the wound which was washed out. Superficial and deep samples were taken and cultured for anaerobic and aerobic bacteria, fungi, and acid-fast bacilli. Dural repair, which had been performed after the initial surgery, was without CSF leakage.

At this time, the differential diagnosis included postneurosurgical wound site infection with seroma and possible epidural abscess related to nosocomial meningitis. Even though CSF by lumbar puncture was not obtained to confirm meningitis (as the patient was taken emergently to surgery), the MRI finding of contiguity between the epidural collection/abscess and the CSF spaces suggested the presence of meningitis. Broad-spectrum intravenous antibiotics with cefepime and vancomycin were started along with steroids while awaiting intraoperative cultures. Cultures, however, returned positive for *Aspergillus fumigatus*.

Treatment was promptly started by initiating intravenous voriconazole loading dose 6 mg/kg, every 12 hours for two doses, followed by maintenance dosing of 4 mg/kg every 12 hours. The empiric antibacterial coverage and steroids were discontinued as no bacteria grew from the cultures. Antifungal susceptibilities were sent for *A. fumigatus* and returned with the following minimum inhibitory concentrations: amphotericin 2 *μ*g/ml, caspofungin 0.125 *μ*g/ml, micafungin ≤ 0.015 *μ*g/ml, itraconazole 0.25 *μ*g/ml, posaconazole 0.06 *μ*g/ml, and voriconazole 0.25 *μ*g/ml (No CLSI interpretation available). Voriconazole was continued, and a treatment duration of nearly five months was completed with an excellent clinical response. A repeat MRI scan of the brain showed no remaining epidural collection with an overall improvement in the patient's clinical status.

## 3. Discussion

*Aspergillus* spp. are opportunistic pathogens, typically causing invasive disease in immunocompromised patients, and represent a rare cause of potentially fatal surgical wound infections and abscesses in affected patients [2, 5, 6, 8, 9]. Much research has associated the risk of postsurgical aspergillosis with the use of corticosteroids, such as daily prolonged prednisone use [2, 5, 10]. Other cases have reported aspergillosis in the nervous system in immunocompetent hosts postsurgery [5, 6, 11, 12]. Our patient did not have any evidence of immunocompromise; her HIV test was negative, and she did not have a history of corticosteroid treatment before surgery.

There are numerous pathogenic *Aspergillus* spp. of which *A. fumigatus* is the most common species isolated postneurosurgery [2, 5, 11]. *A. fumigatus* is ubiquitous in the environment and commonly transmitted through conidial inhalation. Pasqualloto et al. reported 25 cases of aspergillosis due to neurosurgery, and Panicker et al. described 5 others due to combined ear, nose, and throat and neurosurgical procedures [5, 13]. In addition to the inhalational route, the central nervous system (CNS) may also be a destination from hematogenous dissemination or directly spread from nasal or paranasal deposits. We have included a brief literature review of cases found to have *A. fumigatus*-associated postsurgical wound infections in immunocompetent patients (Table 1).

The potential for exposure to *Aspergillus* spp. in the hospital and contamination in the operating room exists, particularly in areas with high traffic of construction/renovation or environmental outbreaks. Infections of the wounds, heart, lung, and CNS have been reported following surgical procedures [5, 19]. Gunaratne et al. even reported an outbreak of *A. fumigatus* from contaminated unused hospital materials (syringes, needles, and cannulae) following a natural disaster [20]. Infection prevention measures that include indoor air sampling, regular ventilation system maintenance, and adequate sterilization techniques are recommended to mitigate the development of these types of nosocomial infections [1, 2, 5, 20, 21].

Diagnosing aspergillosis in the CNS postsurgery can be very challenging. Most patients present with fever and headache followed by neck stiffness many weeks after the procedure, but not all patients follow this pattern [2, 11]. There is no “gold standard” test for the diagnosis of aspergillosis, and various methods are often used, such as culture examination, histopathology, microscopy, cytopathology, fungal biomarkers, needle aspiration or specimen biopsy, and immunodetection [2, 19, 22]. Serum galactomannan testing can be helpful in the diagnosis of CNS infections; however, tests may not always return positive [2]. The national consensus guidelines for the diagnosis of invasive aspergillosis in 2021 mentions galactomannan antigen testing to be one of the most common nonculture methods for diagnosis [22]; however, due to limitations with sensitivity and potential cross-reactions from other fungi or drugs, a negative result should not rule out disease if the overall clinical picture may suggest otherwise [6, 9, 23]. Zamora et al. reported that CSF galactomannan testing was more useful in detecting aspergillosis caused by *A. fumigatus, A. terreus, and A. flavus* [2]. Using samples of 5 patients diagnosed with CNS aspergillosis, Kami et al. reported in 2002 that CSF galactomannan testing had a sensitivity and a specificity of over 80% and 90%, respectively, but fungal cultures were negative for all of them [24]. In our case, serum galactomannan was negative, and CSF galactomannan was never performed due to the lack of validation for this type of test. The diagnosis of *Aspergillus* epidural abscess was made after cultures taken from the operating room grew *A. fumigatus*.

Early diagnosis and treatment of *Aspergillus* surgical wound infections and abscesses can reduce morbidity and mortality [2, 5, 6]. It is also important to have a broad differential when evaluating and managing patients with postsurgical wounds, considering the potential presence of fungi. Data on the treatment of *A. fumigatus* surgical wound infections and abscesses are limited, particularly for immunocompetent patients. A review of the literature supports a combined approach of surgical debridement to remove infected tissue and an effective antifungal [1, 2, 9, 12]. The preferred drug for *Aspergillus* infections due to *A. fumigatus* is voriconazole [1, 6, 22, 25]. Voriconazole exhibits excellent *in vitro* susceptibilities against *A. fumigatus*, ranging from 84.8 to 95.9% [26, 27], and is a favorable drug to treat epidural CNS infections due to its ability to penetrate both the CNS and abscess tissue [1, 6, 8, 11, 22, 28–30]. Other recommended treatment options include amphotericin B, posaconazole, isavuconazole, itraconazole, and echinocandins, although numerous case reports have shown voriconazole to be superior [1, 6, 8, 22, 25]. In a review of 123 cases of CNS aspergillosis, amphotericin monotherapy demonstrated higher mortality than voriconazole (62.5 vs. 35.4%, respectively) [31]. Few studies have also shown that echinocandins used in conjunction with amphotericin B or voriconazole can produce a synergistic effect [21, 32]. Duration of therapy should be driven by the source of infection and can last several months [8, 11]. In our case, antifungal therapy was given for about 5 months and discontinued upon clinical improvement and image resolution.

In conclusion, we have illustrated a relatively rare case of epidural abscess and wound infection following a neurosurgical procedure in an immunocompetent person due to *A. fumigatus*. Diagnosis can be challenging as there is no uniform presentation, and fungal pathogens are often less frequently considered as the causative pathogen in immunocompetent patients. Delayed diagnosis and treatment may lead to poor outcomes; therefore, a high index of suspicion and timely diagnosis, coupled with effective antifungals, are essential in preventing morbidity and mortality in postneurosurgical aspergillosis.

## Figures and Tables

**Table 1:** Literature review of *Aspergillus fumigatus* associated with postsurgical wound or abscess in immunocompetent patients.

Reference	Age/sex	Surgery performed	Interval between surgery and infection	Infectious complication	Method of diagnosis	GM status	Antifungal treatment	Outcome
Index case	54/F	Neurosurgery/suboccipital craniectomy with C1 laminectomy and duraplasty	3 weeks	Wound site infection with seroma and epidural abscess with meningitis	Surgical excision, culture	Negative	Voriconazole (loading dose then 350 mg twice a day) for approximately 5 months	Alive. Resolution of epidural abscess
Chen et al. [9]	46/F	Mastoidectomy/craniectomies/chronic otitis media	Approx. 2 years and 5 months	Otogenic cerebellar abscesses	L/CSF; surgery; pus culture	NA	Voriconazole (400 mg/day) for 5 months	Alive
Morioka et al. [14]	83/M	Neurosurgery/burr hole subdural hematoma	Approx. 3 years and 5 months	Subdural abscess and granuloma	Surgical excision histopathology	NA	L-AMB (0.25 biweekly intrathecally) + miconazole (500 mg IV daily); adverse reaction; replaced by flucytosine (5 g orally daily)	NA
Darras-Joly et al. [15]	29/M	Neurosurgery/acoustic neurinoma	NA	Meningitis; abscess	L/CSF	Positive	L-AMB (5 weeks) + 5-FU (7 weeks); itraconazole (6 months)	Alive after 12 months
Letscher et al. [16]	20/M	Frontal craniectomy/brain trauma	NA	Cellulitis/epidural abscess/frontal bone osteomyelitis	Brain biopsy, culture	NA	ABLC × 3 weeks followed by itraconazole for 5 months	Alive after 3 years
Endo et al. [17]	55/M	Neurosurgery/pituitary adenoma	Approx. 1 year	Arachnoiditis; subdural abscess	L/abscess aspiration	ND	AMB + fluconazole (4 weeks)	NA
Marinovic et al. [18]	65/M	Cranioplasty for severe craniofacial trauma	Approx. 8 years	Meningitis, abscess	L/CSF; stereotactic drainage	NA	AMB; L-AMB; itraconazole	Alive

GM, galactomannan; AMFB, amphotericin; L-AMB, liposomal amphotericin; ABLC, amphotericin B lipid complex; NA = not available; Approx. = approximately.

## Data Availability

All data needed to support the findings are in the manuscript.
